# Highly dynamic wintering strategies in migratory geese: Coping with environmental change

**DOI:** 10.1111/gcb.14061

**Published:** 2018-02-20

**Authors:** Kevin K. Clausen, Jesper Madsen, Fred Cottaar, Eckhart Kuijken, Christine Verscheure

**Affiliations:** ^1^ Department of Bioscience Aarhus University Rønde Denmark; ^2^ Haarlem The Netherlands; ^3^ Beernem Belgium

**Keywords:** land use change, migration, philopatry, pink‐footed geese, site use, waterfowl

## Abstract

When and where to move is a fundamental decision to migratory birds, and the fitness‐related costs and benefits of migratory choices make them subject to strong selective forces. Site use and migration routes are outcomes of opportunities in the surrounding landscape, and the optimal migration strategy may be conservative or explorative depending on the variability in the environment occupied by the species. This study applies 25 years of resighting data to examine development in winter migration strategy of pink‐footed geese divided among Denmark, the Netherlands and Belgium, and analyse potential drivers of strategy change as well as individuals’ likelihood to break with migratory tradition. Contrary with the general notion that geese are highly traditional in their winter site use, our results reveal that winter migration strategy is highly dynamic in this species, with an average annual probability of changing strategy of 54%. Strategy was not related to hunting pressure or winter temperature, but could be partly explained by a tracking of food resources in a landscape of rapid land use changes. The probability of individuals changing strategy from year to year varied considerably between birds, and was partly related to sex and age, with young males being the most likely to change. The annual probability of changing wintering strategy increased substantially from ≈40% to ≈60% during the study period, indicating an increasingly explorative behaviour. Our findings demonstrate that individual winter strategies are very flexible and able to change over time, suggesting that phenotypic plasticity and cultural transmission are important drivers of strategy choice in this species. Growing benefits from exploratory behaviours, including the ability to track rapid land use changes, may ultimately result in increased resilience to global change.

## 1 INTRODUCTION

One of the most fundamental decisions faced by animals is movement in time and space. When and where to move is of utmost importance to everything from food acquisition and avoidance of adverse weather conditions, to finding a mate and avoiding predators, and hence an important determinant of individual fitness (Sharma, Couturier, & Cote, 2009; Swingland & Greenwood, 1983). Nowhere in the animal kingdom is this more apparent than for migrating birds, and the ability to make optimal migratory choices should consequently be subject to strong selective forces (Lameris et al., 2017; Prop, Black, & Shimmings, 2003). General life‐history theory predicts that new traits will only develop if they improve the fitness of the individuals employing them, and that traits are constantly shaped by the environment surrounding the individuals carrying them, by means of evolutionary forces acting to optimize survival and reproduction (Stearns, 1992). The optimal migratory choice, however, may vary depending on the variability in the environment that is occupied, and may change through time by means of extrinsic factors affecting suitability of different areas. At two hypothetical extremes, a stable environment may favour decision‐related traits strengthening site fidelity, while a very dynamic environment may favour traits supporting explorative deeds (Greenwood, 1987). In reality, the optimal level of philopatry is probably often somewhere in‐between, allowing for both knowledge of previously exploited areas and the necessary flexibility in site‐choice to cope with changing environmental conditions.

Among waterbird species, an often reported feature in relation to migration is site‐faithfulness (also termed philopatry). As such, migratory waterbirds have been found to exhibit philopatry to their breeding (Blums, Nichols, Hines, & Mednis, 2002), staging (Kruckenberg & Borbach‐Jaene, 2004) and wintering sites (Robertson & Cooke, 1999). Individual sites may be beneficial in terms of food abundance, finding a mate, perceived predation risk (or disturbance levels) or any combination of these (Anderson, Rhymer, & Rohwer, 1992; Robertson & Cooke, 1999). Knowledge of where to go might also be an advantage to species relying on specific habitats with patchy distributions by minimizing the costs of movement and resource assessment (Cooch, Jefferies, Rockwell, & Cooke, 1993; Robertson & Cooke, 1999; Rohwer & Anderson, 1988).

When site‐fidelity operates year‐round on the geographical scale of population flyways, the recurrent use of the same staging sites outside the breeding season emerges into “wintering strategies” of site‐faithful birds. Like their fidelity to single sites, waterfowl species are generally believed to be faithful to their wintering strategies, visiting and wintering in the same localities year after year (Fox et al., 1994). Indeed, the consistent population‐specific patterns of breeding, migration and choice of wintering area is what underlie the concept of waterfowl population flyways (Boere & Stroud, 2006). Faithfulness to sites or strategies is a feature of individuals, not populations, and the degree of fidelity may vary among age classes, sexes and personalities of individual birds. Rarely however, has the philopatric nature of different demographic groups been put to the test, and rarely are the time‐series of data long enough to follow how philopatry may change in bird populations over time. Also, the number of individually marked and observed birds is seldom large enough to derive conclusions on the nature of philopatry in entire species.

In this study, we use 25 years of mark‐resight data covering >4,000 individually neck‐collared pink‐footed geese (*Anser brachyrhynchus*) and >370,000 resightings to test the faithfulness of individual geese to their wintering strategy. Specifically, we aim to (i) Describe the development in wintering strategy for this population during the period 1990–2015, (ii) Investigate how propensity to change strategy has developed during the last 25 years, (iii) Explore the effects of age, sex, breeding status and previous migratory choices on the tendency to change strategy, and (iv) Test whether strategy choice was related to the environmental factors hunting pressure, winter temperature and land use. In this connection we hypothesized that (i) Increasing hunting pressure in one region would cause birds to depart from this area, (ii) Cold winters would cause a southward shift in wintering site use and (iii) Changes in the available area of favoured crops, such as cereals and maize, would cause geese to gather in regions of high food availability.

## 2 MATERIALS AND METHODS

### 2.1 Focal population

Svalbard‐breeding pink‐footed geese migrate via Norway to wintering areas in Denmark, the Netherlands and Belgium (Madsen, Cracknell, & Fox, 1999). The population has been steadily increasing over the last decades and currently totals around 75,000 birds (Madsen, Cottaar et al., 2016). During the last decades, wintering pink‐footed geese have increasingly abandoned marshes and pastures in favour of agricultural habitats, and now forage almost exclusively on energy rich agricultural crops such as waste grain and maize cobs on stubble fields, newly sown winter cereals, newly sown spring cereals and cultural grasslands (Fox et al., 2005; Madsen, Christensen, Balsby, & Tombre, 2015). Nights are spent on large disturbance free water bodies, isolated islets in coastal areas or in wet marshes. Like most waterfowl species pink‐footed geese are relatively long‐lived (average life span of 5–6 years, oldest known individual >22 years). They mature and mate at an age of c. 3 years, and are monogamous with paired adults staying together year‐round. Young birds follow their parents in the first autumn and winter.

This population of pink‐footed geese has been subject to a long‐term ringing scheme initiated in 1990. Since then, >4,000 individual geese have been captured and marked with metal rings and plastic neck‐collars (Table 1). Most birds have been captured with cannon nets in spring (March‐May) in western Jutland (Denmark) and Nord‐Trøndelag (Norway), but a minority (<500) have been caught and marked by rounding up geese during wing moult on the Svalbard breeding grounds. At capture, geese were sexed by cloacal examination and aged by feather characteristics. The presence of the neck‐collars does not seem to have any long‐term negative effects on body condition of the geese (Clausen & Madsen, 2014).

**Table 1:** Annual ringing effort (number of birds) and sample sizes (number of individuals with observations) to infer proportional use of the seven wintering strategies in the period 1991–2015

Year	Ringing effort	Sample size
1990	97	
1991	166	249
1992	151	368
1993	4	323
1994	100	357
1995	131	440
1996		343
1997		300
1998	339	540
1999		445
2000	151	514
2001	192	640
2002	274	746
2003	205	872
2004	289	923
2005	395	1148
2006		910
2007	538	1350
2008	105	1162
2009	189	1139
2010		709
2011	168	704
2012	232	804
2013		555
2014	37	418
2015	381	619
Sum	4144	16669

### 2.2 Definition of wintering strategies

To infer the whereabouts of individual birds, we used resightings of marked geese across their entire wintering range. Throughout the years, a comprehensive and consistent resighting effort of neck‐collared birds has been assured by systematic resighting campaigns carried out by trained observers during the nonbreeding season (in Denmark, the Netherlands and Belgium). To gather as many resightings as possible the systematic effort was supplemented by voluntary observations (all data reported online to the internet portal http://www.geese.org). The number of professional observers was relatively constant throughout the years, ensuring good coverage across the entire study period. However, total observer numbers varied in response to the volume of amateur contributions (Figure S1). Individual birds were seen several times each winter (mean ± *SE*: 21.3 ± 0.13), and observation effort was very constant across the entire study period, with the average annual number of observation days being 198 days (*SE* = 3.2, Figure S2). Obvious misreadings of neck‐collars (e.g. combinations that did not exist) were omitted from our data set, but occasional none‐obvious misreadings may occur from professionals and amateurs alike. However, with the large amount of data available sporadic misreadings will only have negligible effects.

**Figure 1:**
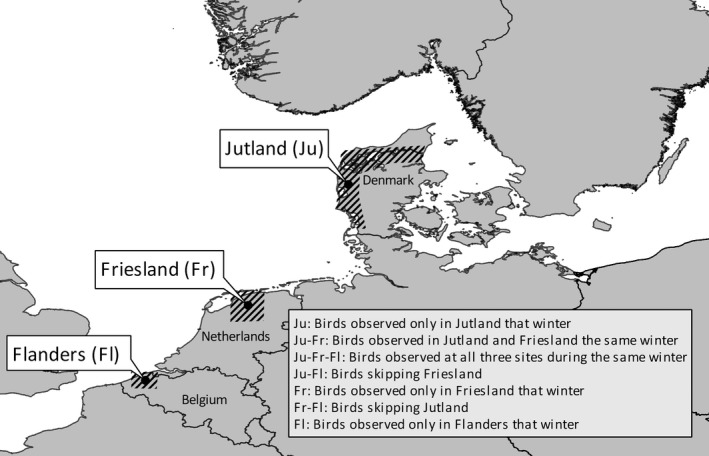
The three stopover regions and seven wintering strategies (text box) used by pink‐footed geese

Dealing with observational data from a species with a highly mobile foraging activity, has certain limits to the spatial resolution of our defined “sites”. Making observations at the night roosts is not an option, and the highly variable use of the surrounding landscape by pink‐footed geese render small‐resolution analyses very prone to false negatives (not identifying a bird that was actually present at some point during the nonbreeding season). As a consequence, we restricted our choice of sites to three major and well‐separated regions used by pink‐footed geese in the wintering period: Jutland (Denmark), Friesland (the Netherlands) and Flanders (Belgium, Figure 1). This delineation of staging sites excludes inference of local site fidelity, but ensures a much more reliable measure of presence/absence of individual birds within each overall stopover site. We defined a wintering strategy as the annual subset of these three regions used in the entire period from when geese arrive from Norway to these areas (late September to early November) until when they start to leave on spring migration (early April to early May). Collectively, the possible combinations of these three major stopover sites gave rise to seven different wintering strategies (defined in Figure 1).

Annual use of wintering strategies was inferred from the proportion of all marked birds using each of the seven strategies in any given year. Birds were ringed in spring by the end of a wintering season, and the incomplete data from geese in their ringing season were excluded from analyses. The first year of the time series (1990) was omitted due to a relatively low sample size. In all the analyses below “Year” indicates the starting year of a wintering season (e.g. 1991 is the 1991/1992 winter).

### 2.3 Changes in population wintering strategy

Potential trends and changes in proportionate use of the seven wintering strategies was assessed using piecewise linear regression to objectively identify inflection points across the 25 year period. Changes in slope were tested using the Davies test (Davies, 2002), and the analyses conducted using the R package “segmented” (Muggeo, 2008). Based on the inflection points from this analysis, we divided the changes in wintering strategy into four different periods (1991–1996, 1997–2006, 2007–2010 and 2011–2015; see 3). To identify directional movements between the different strategies in each of these periods, we identified all events of birds changing strategy and calculated the pairwise net exchange between strategies in proportion to all strategy changes within the given period. The net exchange was visualized using the package “diagram” (Soetaert, 2009), and small exchanges below 2.5% of all changes in any period were omitted for clarity. This, and all subsequent statistical analyses, were done in R (R Development Core Team, 2008).

### 2.4 Changes in faithfulness to wintering strategy and the role of density dependence

To investigate the development in faithfulness to wintering strategy we identified all events with year‐on‐year data from the same marked bird, and assigned it as either changing strategy (using different subsets of the three major staging regions in two subsequent years) or not changing (using the same regions in subsequent years). Due to a continuously increasing population size throughout most of our study period, the explanatory variables time (years) and population size (both continuous) were highly correlated (pearson's *r* = .95) and causally inseparable. As a consequence only “Year” was included in our model, which may then represent the combined effects of a change in behaviour over time and a potential influence of density dependence on the probability to change strategy. The annual probability of changing was modelled using mixed effects logistic regression with a binomial response variable (changing, not changing), “Year” as a fixed effect and a logit link function (Nelder & Wedderburn, 1972). “BirdID” (identifier of individually marked birds) was included as a random effect to account for individual differences in the tendency to change strategy. The generalized linear models in this and subsequent sections were fitted using the packages “lme4” (Bates, Maechler, Bolker, & Walker, 2014) and “effects” (Fox, 2003).

### 2.5 Effects of sex, age and breeding status on faithfulness to wintering strategy

The tendency of different sex and age groups to change wintering strategy was modelled using the same binomial response (changing vs. not changing). Differences between sex and age groups was investigated using a generalized linear mixed model with “Sex and age group” as a fixed effect, “Bird ID” and “Year” as random effects accounting for individual and annual differences in the tendency to change, and a logit link function. Because birds were marked by the end of their first winter, and we only had full wintering data from their second winter forth, the first potential change in strategy that could be acknowledged from these data was when birds were in their third winter. Based on this reflection we defined the four age and sex groups 3rd winter males, 3rd winter females, adult males and adult females.

To assess whether a change in breeding status could influence the tendency to change strategy we ran a generalized linear mixed model with “Breeding status” as the fixed effect and “Bird ID” and “Year” as random effects. In terms of breeding status we only distinguished between individuals accompanied by young on autumn migration (successful breeders) and individuals not (nonbreeders, unsuccessful breeders). “Breeding status” was defined by the two levels “same” and “different”, indicating whether an individual had the same breeding status in two consecutive years or not. The presence of young was recorded by trained observers during the systematic resighting campaigns in autumn. To avoid pseudoreplication only one individual from a pair was included in the analysis.

### 2.6 Individual differences in the likelihood to change strategy

In order to assess individual differences in the likelihood to change strategy, and whether some birds were more likely to change than others, we identified all immediately successive wintering events and asked whether an individual changing strategy in 1 year (year *t*) was more likely to change again in the following year (year *t + 1*). The event in “year *t + 1”* (changing or not) was modelled as a binomial response variable using a generalized linear mixed model with logit link. The event in “year *t”* (changing or not in previous year) was included as a fixed effect, and to account for potential confounding effects of individual and annual differences, “Bird ID” and “Year” were included as random effects. The response variable in this analysis was identical to the response described above, but the data set was smaller as this analysis was conditional on data from three consecutive years.

### 2.7 Effects of hunting pressure, winter temperature and land use

As a measure of hunting pressure in Denmark (pink‐footed geese do not have an open season in the Netherlands and Belgium) we used the annual harvest rate (proportion of the population being shot, Clausen, Christensen, Gundersen, & Madsen, 2017). Average annual winter temperatures (Dec–Feb) were acquired from weather stations in Esbjerg (Jutland), Leeuwarden (Friesland) and Oostende (Flanders), representing each of the three major stopover regions. These data were downloaded from the National Climatic Data Centre (NCDC) of the National Oceanic and Atmospheric Administration (NOAA, https://www7.ncdc.noaa.gov/CDO/cdoselect.cmd). Investigating the occurrence of severe cold spells as explanatory variable revealed that these only occurred in already cold winters, indicating that most explanatory power was captured by average temperature alone. Agricultural land use data (area of different crops) from North and West Jutland (core staging areas in Jutland), Friesland and Flanders, respectively, were made available by Statistics Denmark, Statistics Netherlands and The Government of Flanders’ Department of Agriculture and Fisheries. From these data we extracted the area of arable land covered by food crops used in the three regions. These include spring cereals (in autumn providing spilled grain on stubble fields), winter cereals (newly sprouted plants), maize (spilled cobs on stubble fields), grasslands, and in Flanders also potatoes (leftovers on harvested fields) (Cottaar, 2009; Kuijken & Verscheure, 2016; Madsen et al., 2015). Due to changes in the administrative units of Denmark in 2007, the geographical units of statistical data gathered before and after this year were slightly different. In order to ensure a continuous data series of fixed area, the larger areas of crops in the new units of West and North Jutland were downscaled in proportion to the difference in total arable land, by multiplying with a fraction calculated as the area of arable land in the new units divided by the area of arable land in the old units. In 2012 data were missing for parts of Jutland, and as a consequence this year was omitted from the land use analysis in Denmark. The data on hunting pressure, winter temperature and land use are presented in Figures S3‐S5.

From the nonindependence of our seven wintering strategies (proportions must sum to 1) it follows that causal relationships with hunting pressure, winter temperature or area of available foraging habitat in any part of the flyway will also affect proportionate use of the other sites (and hence strategies).

Acknowledging this fact, we approached the analysis of potential driving factors by first examining what factors might have affected the proportions of birds staying in Jutland ‐ the first wintering site encountered by most geese on southward migration. Choices made on this first stopover (to stay or not) would invariably entail changes in the proportion of birds moving further south. Next, we investigated changes in the use of Friesland and Flanders, respectively, by summing the proportions of birds across all the strategies where the given region was used. Relationships between proportions of birds (dependent variable) and hunting pressure (Jutland), winter temperature (all three regions) and land use (all three regions) were analysed by means of general linear models. All models tested for both linear and quadratic effects.

## 3 RESULTS

### 3.1 Changes in population wintering strategy

**Figure 2:**
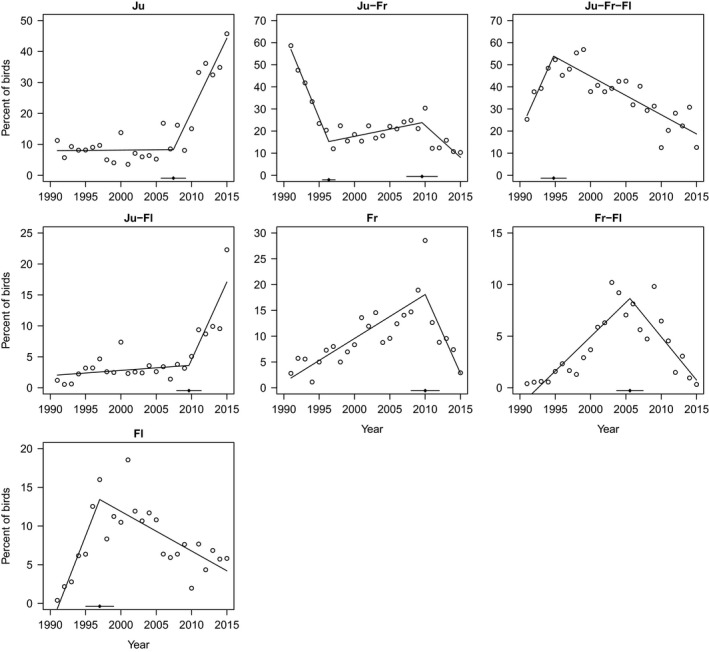
Proportionate use (% of total population) of the seven wintering strategies of pink‐footed geese during the period 1991–2015. Solid lines indicate the best fit from piecewise linear regression, and points along the *x*‐axis the inflection points (with 95% confidence limits)

Annual sample sizes to clarify prevalence of, and changes in, wintering strategy ranged from 249 birds in 1991 to 1350 birds in 2007 (Table 1). The proportion of birds staying in Jutland was relatively constant around 8% up until 2007, and showed a rapid increase in recent years to reach 46% in 2015 (Figure 2). In the first years of the study, the most prominent migration strategy was Jutland‐Friesland (≈60% in 1991). The proportion using this strategy showed a decline during the early 1990s to ≈11% in 1997, a slightly increasing trend from 1997 to around 2010, and further decrease thereafter. The percentage of geese using all three stopover sites increased initially from 25% to just over 50%, but has fallen since to reach only 12% in 2015. The proportion of birds skipping Friesland (observed in Jutland and Flanders) was relatively stable below 5% up until 2010, and then rose rapidly to reach just above 20% in 2015 (Figure 2). The proportions observed only in Friesland, and only in Friesland and Flanders, both showed unimodal developments peaking around 2010 and 2005, respectively, at proportions of ≈20% and 10% (Figure 2). Finally, the percentages observed only in Flanders increased from below 5% in the early 1990s to reach approximately 15% around 2000, and have since declined to c. 5% in recent years.

**Figure 3:**
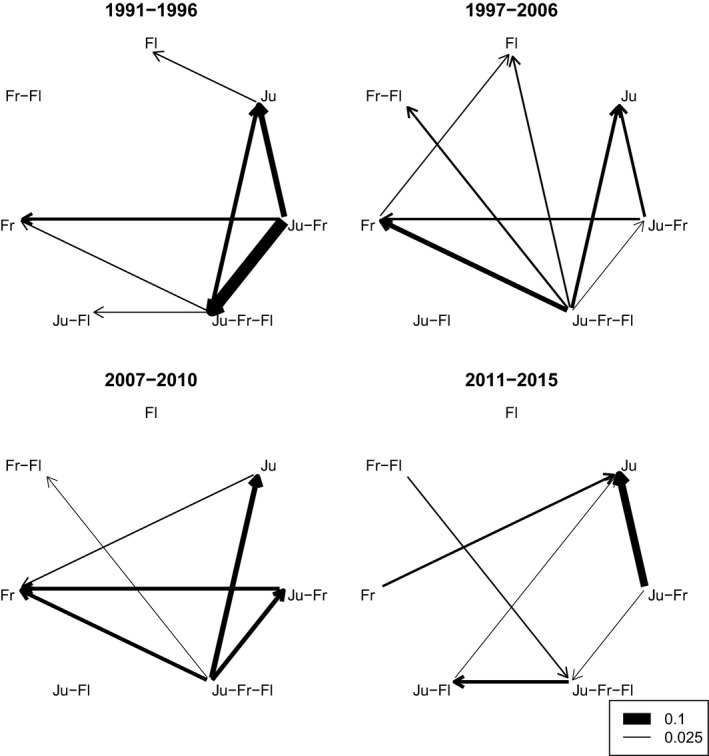
Net exchange rates (proportion of total changes in each time period) between the different wintering strategies used by pink‐footed geese. The four time periods were defined from the break‐points identified by piece‐wise linear regression in Figure 2. Small net exchanges below 2.5% were omitted for clarity

Net exchange rates between the different strategies (available in Table S1) revealed that during the period 1991–1996 birds generally abandoned the Ju‐Fr strategy in favour of either continuing south to include Flanders, staying in Jutland all winter or going directly to Friesland (Figure 3). During 1997–2006 there was a strong movement away from the Ju‐Fr‐Fl strategy to all the others, with most changing birds shifting to the Fr strategy. In 2007–2010 the increase in proportions using Ju, Fr and Ju‐Fr were fuelled by birds shifting away from the Ju‐Fr‐Fl strategy (a decline in the use of Flanders), and in 2011–2015 the strong increases in the use of Jutland and to some extent Flanders (Ju, Ju‐Fl) were driven by a strong decline in the birds’ use of Friesland (Figure 3).

### 3.2 Changes in faithfulness to wintering strategy and the role of density dependence

In total 10,849 events of year‐on‐year data from the same bird were identified, covering 2,941 individually different birds. The annual probability of changing wintering strategy increased linearly during the study period (*z* = 10.760, *p* < .001) from ≈0.4 in the early 1990s to ≈0.6 in recent years, indicating that birds have become increasingly more exploratory during this period.

### 3.3 Effects of sex, age and breeding status on faithfulness to wintering strategy

**Figure 4:**
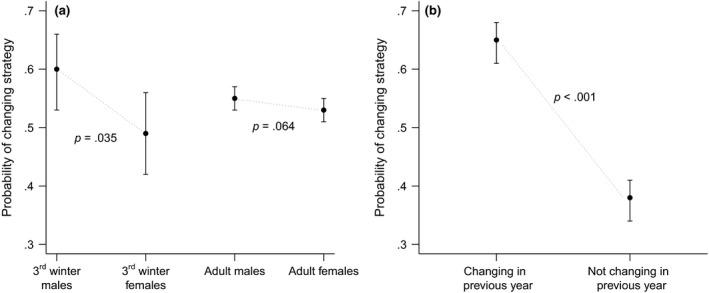
The likelihood of changing strategy in any given year for each of the four sex and age groups distinguished in this study (a), as well as the probability of changing strategy in relation to choice (to change or not) in previous year (b). *p*‐values and 95% confidence intervals are shown

Across the four sex and age groups the likelihood to change strategy in any given year ranged between 0.60 in 3rd winter males to 0.49 in 3rd winter females (Figure 4a). Pairwise comparisons between the sexes revealed that among 3rd winter birds males were significantly more likely to change than females (*z* = −2.111, *p* = .035), a relationship not significant for adult birds (*z* = −1.854, *p* = .064).

Data on breeding status of individual birds in two consecutive years was available on 2,736 occasions throughout the study period, covering 1,238 different birds. Breeding status did not affect the probability of changing strategy (*z* = 0.205, *p* = .838), indicating that birds with the same breeding status in two consecutive years were just as likely to change strategy as birds changing breeding status in the same period.

### 3.4 Individual differences in the likelihood to change strategy

The number of immediately successive events of changing (or not) amounted to 7,646 records from 2,254 different birds. The likelihood to change strategy in any given year was significantly affected by the event of the previous year, so that birds changing strategy in year *t* were also more likely to change again in year *t + 1* (Figure 4b).

### 3.5 Effects of hunting pressure, winter temperature and land use

The proportions of birds staying in Jutland were positively linearly associated with harvest rate (*N* = 25, *F* = 31.246, *p* < .001), thereby ruling out hunting pressure or hunting induced disturbance as an important driver of strategy choice on a flyway scale. Local winter temperature did not significantly affect proportions of birds staying in Jutland (*N* = 25, *F* = 0.977, *p* = .333) or proportions of birds using Friesland (*N* = 25, *F* = 1.704, *p* = .205) and Flanders (*N* = 25, *F* = 0.208, *p* = .653). None of the quadratic terms were significant either.

The data on land use in Jutland indicated a substantial increase in the area of maize and a decline in the area of spring cereals. In Friesland noteworthy changes were a unimodal pattern in the area of maize (peaking in 2008) and a slight decrease in the area of grassland. Flanders experienced a considerable drop in grassland area (especially from 2000 onwards) and an increase in the area of maize (Figure S5). The land use analysis revealed that proportions staying in Jutland were positively linearly associated with the area of maize, while usage of Friesland and Flanders were positively linearly associated with the area of grasslands (Table 2). From Figure 2 it follows that the large increase in the use of the Ju strategy after 2007 more or less counterbalanced the drop in remaining strategies during the same period. Because many geese initially arrive in Jutland during autumn, before migrating further south, the recent drop in wintering strategies including Friesland and Flanders might therefore first and foremost relate to a positive selection of Jutland rather than a deselection of these areas. A strong indication of this was seen from a significant quadratic term between area of maize and proportions of geese in Flanders (*p* = .002). Proportions of geese using Flanders showed a positive association with the area of maize up until ≈2007, after which a continuous rise in maize area was associated with a drop in goose proportions. Given the otherwise strong positive association with area of maize, this indicated that “short‐stopping” in Jutland could lead to an uncoupling of the effects of land use changes further south. To acknowledge this fact, we re‐ran the models for these two regions omitting the years from 2007 onwards. This procedure indicated that usage of Friesland was unaffected by land use changes, while usage of Flanders was linearly positively related to the area of maize and grassland (Table 2). None of the quadratic terms were significant in these final models.

**Table 2:** Scaled estimates (estimate ± *SE* (*p* value)) from the general linear models describing strategy choice (proportions of the population staying in Jutland, using Friesland and using Flanders) in relation to changes in land use within each region during the period 1991–2015

	Proportion staying in Jutland	Proportion using Friesland	Proportion using Flanders	Proportion using Friesland (< 2007)	Proportion using Flanders (< 2007)
Spring cereal	−0.071 ± 0.065 (0.290)	0.012 ± 0.097 (0.901)	0.021 ± 0.126 (0.867)	0.024 ± 0.058 (0.675)	0.068 ± 0.088 (0.454)
Winter cereal	**−0.153 ± 0.060 (0.020)**	−0.015 ± 0.156 (0.922)	0.015 ± 0.143 (0.919)	−0.011 ± 0.062 (0.858)	0.123 ± 0.118 (0.323)
Maize	**0.103 ± 0.024 (<0.001)**	0.092 ± 0.139 (0.516)	0.240 ± 0.145 (0.115)	−0.096 ± 0.067 (0.182)	**0.303 ± 0.086 (0.006)**
Grassland	0.069 ± 0.036 (0.072)	**0.267 ± 0.079 (0.003)**	**0.253 ± 0.077 (0.004)**	−0.044 ± 0.056 (0.443)	**0.153 ± 0.052 (0.015)**
Potatoes	**‐**	‐	0.021 ± 0.061 (0.730)	‐	0.042 ± 0.043 (0.353)

Estimates are scaled to have a mean of zero and a range from −1 to 1. Bold text indicates significant effects on α‐level 0.05. *N* = 25 years for Friesland and Flanders, and 24 years for Jutland, were 2012 was omitted due to the lack of land use data (see Methods). Because none of the quadratic terms were significant in the final models only linear effects are shown for simplicity.

## 4 DISCUSSION

Our results illustrate that choice of wintering strategy among pink‐footed geese is flexible and dynamic. The proportion of the population using each strategy changed markedly across our 25 year study period, and on average more than half of all individuals changed strategy in any given year. These results are of interest, given the high levels of site‐fidelity waterfowl are believed to have. Indeed, we found a high probability of individuals changing strategy between years (varying between 0.49 and 0.60 among age and sex classes). This probability also increased over time. Our analysis demonstrated that changes in wintering dynamics were not merely the result of turnover of individual birds with individual preferences, but to a large extent the result of individuals changing strategy between years. The social structure of geese is very complex (Lamprecht, 1986), and choice of migration routes and winter site use is probably driven by a minority of individuals with explorative personalities, magnified manifold by the influence of these individuals on conspecifics copying their choice. Such cultural transmission between individuals is probably a common phenomenon among migrating social birds, and has previously been shown for barnacle geese *Branta leucopsis* (Jonker et al., 2013) and whooping cranes *Grus americana* (Mueller, O'Hara, Converse, Urbanek, & Fagan, 2013). The fact that birds changing strategy in 1 year were much more prone to do so again, strongly support an individual component in the tendency to change strategy – a feature not fully explained by sex, age and breeding status. This “exploratory” feature of some birds, where individuals switch between strategies in response to previous experience, suggests that geese explore environmental conditions to adopt an optimal migration strategy (Madsen, 2001). The degree of exploration might be driven by demography (e.g. age, breeding status), past life‐history events (disturbances, competition etc.) or specific genetic features and merits further investigation.

The increasing probability of changing wintering strategy during the last 25 years might relate to a growing impact of intra‐ and interspecific competition on the wintering grounds, but might also be the result of increasing benefits from an exploratory behaviour among individual geese in a time of global change. The foraging opportunities (Madsen et al., 2015), roosting possibilities (Clausen & Madsen, 2016), spring weather conditions (Tombre et al., 2008) and hunting pressure (Clausen et al., 2017) experienced by pink‐footed geese have all changed rapidly recently, and might increasingly favour exploratory traits over traditional philopatric ones. Further evidence of recent exploratory behaviours can be seen from an expanding distribution into Sweden and Finland (Madsen, Cottaar et al., 2016), and the use of new staging areas in Jutland (Madsen et al., 2015). In Friesland and Flanders, however, the population is still concentrated inside relatively small and traditional areas (Kuijken & Verscheure, 2016).

The difference in probability of changing between third winter males and females might relate to pair‐formation among this group. Pink‐footed geese generally form pairs when 2–3 years of age, and because waterfowl species are characterized by high levels of female‐biased philopatry, the male is likely to follow his mate's choices after a pair has been formed (Rohwer & Anderson, 1988). In continuation of this, the sex‐specific difference was much less in adult birds that are assumed to have developed long‐term pair‐bonds with partners with whom they travel together.

As explanatory variables of changes in site use both hunting pressure (positively associated with wintering proportions in Denmark) and winter temperature (insignificant for all three regions) were weak predictors, indicating that neither was very important in shaping strategy choice. Harvest rate has increased substantially in Jutland recently (Clausen et al., 2017), and hunting has been used to control numbers as part of an international adaptive management strategy to stabilize population size (Madsen, Clausen, Christensen, & Johnson, 2016). However, this does not seem to have had any negative effect on goose usage of this region. Other studies have found hunting to affect both activity patterns, local site use and body composition of quarry species (Madsen & Fox, 1995; Pearse, Krapu, & Cox, 2012), but hunting‐related movements across large geographical scales have rarely been explored. One explanation of our findings might be that the area used by pink‐footed geese in Jutland has expanded dramatically during the last 10–15 years (Madsen et al., 2015). As a consequence, geese might be able to find suitable, undisturbed foraging sites with low perceived risk from hunting despite an increase in overall harvest. In relation to this, the effect of hunting pressure might still affect distribution locally, although an effect on overall strategy choice was absent in this study.

Winter harshness has historically been found to affect body condition (Clausen, Madsen, & Tombre, 2015) and the use of Jutland as a wintering site among pink‐footed geese (Madsen, 1980). During the 1980s and 1990s, however, growing numbers have overwintered in Denmark despite incidents of cold spells ‐ at least partly due to an increasing use of winter wheat providing nutritious food even at temperatures below the freezing point (Therkildsen & Madsen, 2000). Improved foraging opportunities might thus mitigate bad weather conditions. This study indicates that currently temperature does generally not dictate winter site use of pink‐footed geese in Jutland, Friesland and Flanders, but some threshold level of heavy snowfall probably still exist where food availability in the northern parts of the wintering range becomes so low that geese move further south. To this end, movements of birds from Flanders and Friesland to Jutland during severe weather in December has been observed to result in additional southward movements and presumably elevated energetic costs (Madsen, 1980).

We found indications that land use changes have played a part in explaining strategy choice of pink‐footed geese. In particular, the abrupt increase in proportions staying in Jutland during the entire winter in recent years seemed to coincide with increased areas of maize in this region. In Jutland, maize cultivation has expanded greatly recently in response to a warming climate (Odgaard, Bocher, Dalgaard, & Svenning, 2011). Maize crops are harvested approximately 3 months later than most cereal fields, and the emergence of this food resource later in autumn when most spilled grain on cereal stubble have been depleted or ploughed, probably serves to extend the period of high quality food availability in this region. Increasing use of maize by pink‐footed geese in Jutland has been described by Madsen et al. (2015), and it seems likely that the declining use of Flanders, and especially Friesland, in recent years might be driven by improved late‐autumn food availability further north. This has led to a reduction in migration distance between breeding and wintering areas. Visser, Perdeck, Van Balen, and Both (2009) recently showed that global warming‐induced declines in migration distance is a common feature of many bird species in Northwest Europe. This study indicates that a climate‐induced increase in late autumn food availability at higher latitudes might contribute to the same pattern, at least for birds exploiting agricultural fields as foraging habitats. In Flanders, and to some extent Friesland, our analysis indicated a positive association between goose proportions and grassland areas. Grasslands are still the most important food resource in both these regions (Cottaar, 2009; Kuijken & Verscheure, 2016), and a positive association could therefore be expected. Intensification of agriculture is an often‐reported driver of growing goose populations in Europe and North America (Fox & Abraham, 2017; Gauthier, Giroux, Reed, Bechet, & Belanger, 2005), and the ability of agricultural changes to drive changes in site use is therefore not surprising. Nonetheless, land use changes are probably unlikely to be the sole driver of the annual 54% probability of changing strategy found in this study (average across sex and age classes). Additional external and intrinsic drivers not included in this study are likely to also shape site use and strategy choice of these migratory birds. As such, interspecific competition, disturbance, predation, body condition and social status may all be contributing factors.

Jonker et al. (2013) recently showed that barnacle geese were able to break with migratory traditions in connection with switches between different populations, and our study reveals that migration patterns may also change in response to environmental change. An important conclusion from both studies is that we need to abandon the view of static wintering strategies and migratory traditions among waterfowl species, and acknowledge that migration routes, stopover sites – and ultimately population flyways – are dynamic systems evolving over time. Migratory geese are subject to rapid changes in their environment, and especially in their foraging habitats. The transition to an agricultural habitat use might have released several goose species from their previously rather limited selection of suitable wetland habitats (Fox & Abraham, 2017), thereby lowering the benefits of philopatry during winter. Evolving dynamic wintering strategies may benefit geese as well as other avian species in an increasingly dynamic landscape, making them resilient against environmental change at local as well as flyway scales. The potential for rapid spread of changing traditions is probably conditional on the ability to culturally transmit beneficial traits, and the continuous interactions and cognitive abilities of highly social species may therefore give them an advantage in relation to changing environmental conditions compared to other avian groups (Teitelbaum et al., 2016). Indeed, Mueller et al. (2013) found that for whooping cranes social learning was more important than genetic relatedness in controlling migratory performance. As a result, the ability to adapt may vary considerably among species, and in a time of rapid environmental change the challenge is to distinguish between groups very susceptible to these changes and groups likely to cope. Our study indicates that pink‐footed geese (and probably similar species) are currently able to cope with rapid environmental changes. Whether these changes are purely the result of phenotypic plasticity or underpinned by genetic adaptation is yet to be explored, but could be an interesting next step to improve our understanding of animals’ responses to current global change.
