# Supplementary material for: Highly dynamic wintering strategies in migratory geese: Coping with environmental change
Source: Glob Chang Biol. 2018 Feb 20;24(7):3214–25. doi: 10.1111/gcb.14061 (PMC6032841; doi:10.1111/gcb.14061)
Supplement: Supplementary file 1 [file GCB-24-3214-s001.docx]

**Supplementary appendix**

**Figure S1. Number of observers.** Development in the number of observers (professionals and amateurs) contributing with resightings through the years. Although the number of professional observers was relatively constant throughout the period, total observer numbers varied in response to the volume of amateur contributions. However, the many resightings of individual birds (on average > 21 times per winter) meant that variation in the number of amateur observers had no impact on the analysis of proportional use. As such, there was no significant relationship between proportional use and observer numbers in any of the three sites (P values ranging from 0.08 to 0.32). In support of this, two years with exceptional high observer numbers in Friesland (2009 and 2013) was not reflected in the proportional use of this site, and the recent substantial increase in the use of Jutland was not related to the number of observers there (compare with Fig. 2 in manuscript). The increasing numbers of observers in Flanders during the early 90s probably reflect a growing influx of birds (from a few thousands to > 30,000) to this site that was established as an important wintering area during the very same years (Madsen *et al*., 1999). Labels on the x-axes (years) indicate the starting year of the corresponding winter (e.g. 2000 specify the wintering period 2000/2001).


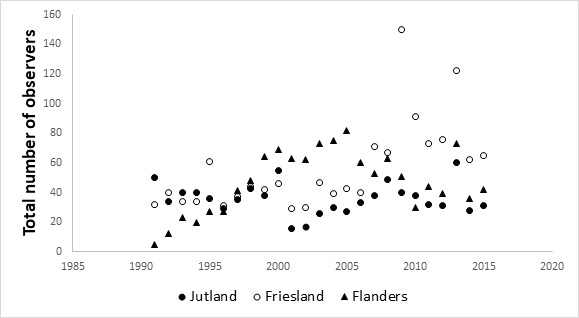


**Figure S2. Resighting effort.** Development in the number of days with observations of neck-collared pink-footed geese from the wintering areas throughout the study period. Equation indicate the best linear fit, supporting the assumption of a relatively constant resighting effort. Labels on the x-axes (years) indicate the starting year of the corresponding winter (e.g. 2000 specify the wintering period 2000/2001).

**Figure S3. Hunting pressure.** The labels on the x-axes (years) indicate the starting year of the corresponding winter (e.g. 2000 specify the wintering period 2000/2001).

**Figure S4. Winter temperature.** The labels on the x-axes (years) indicate the starting year of the corresponding winter (e.g. 2000 specify the wintering period 2000/2001).

**Figure S5. Land use changes.** The labels on the x-axes (years) indicate the starting year of the corresponding winter (e.g. 2000 specify the wintering period 2000/2001).

**Table S1. Net exchange rates.** The figures indicate the proportional exchange between two strategies in relation to all changes during each of the four time periods (each table sums to 1). Please note that figure 3 in the manuscript shows net exchanges (birds can move in both directions).

| **1991-1996** | **Ju** | **Ju-Fr** | **Ju-Fr-Fl** | **Ju-Fl** | **Fr** | **Fr-Fl** | **Fl** |
| --- | --- | --- | --- | --- | --- | --- | --- |
| **Ju** | - | 0.020168 | 0.006723 | 0.001681 | 0.011765 | 0.003361 | 0.026891 |
| **Ju-Fr** | 0.058824 | - | 0.339496 | 0.010084 | 0.038655 | 0.008403 | 0.016807 |
| **Ju-Fr-Fl** | 0.047059 | 0.235294 | - | 0.02521 | 0.026891 | 0.016807 | 0.021849 |
| **Ju-Fl** | 0.003361 | 0.001681 | 0.008403 | - | 0 | 0.003361 | 0.005042 |
| **Fr** | 0.021849 | 0 | 0.005042 | 0.001681 | - | 0 | 0.006723 |
| **Fr-Fl** | 0 | 0 | 0 | 0.001681 | 0 | - | 0.005042 |
| **Fl** | 0.006723 | 0 | 0 | 0.005042 | 0.008403 | 0 | - |
|  |  |  |  |  |  |  |  |
| **1997-2006** | **Ju** | **Ju-Fr** | **Ju-Fr-Fl** | **Ju-Fl** | **Fr** | **Fr-Fl** | **Fl** |
| **Ju** | - | 0.015801 | 0.013093 | 0.004515 | 0.01219 | 0.002709 | 0.017156 |
| **Ju-Fr** | 0.037472 | - | 0.133183 | 0.013544 | 0.034312 | 0.018059 | 0.019413 |
| **Ju-Fr-Fl** | 0.041535 | 0.153499 | - | 0.029797 | 0.051919 | 0.075847 | 0.029797 |
| **Ju-Fl** | 0.006772 | 0.008126 | 0.026637 | - | 0.006321 | 0.00316 | 0.009481 |
| **Fr** | 0.013544 | 0.018059 | 0.010384 | 0.004063 | - | 0.009932 | 0.025734 |
| **Fr-Fl** | 0.003612 | 0.012641 | 0.043792 | 0.00316 | 0.01851 | - | 0.013995 |
| **Fl** | 0.016704 | 0.003612 | 0.004515 | 0.00316 | 0.02167 | 0.008578 | - |
|  |  |  |  |  |  |  |  |
| **2007-2010** | **Ju** | **Ju-Fr** | **Ju-Fr-Fl** | **Ju-Fl** | **Fr** | **Fr-Fl** | **Fl** |
| **Ju** | - | 0.056529 | 0.023742 | 0.009045 | 0.027134 | 0.005653 | 0.006783 |
| **Ju-Fr** | 0.063878 | - | 0.080837 | 0.010175 | 0.072357 | 0.016393 | 0.010175 |
| **Ju-Fr-Fl** | 0.055399 | 0.129452 | - | 0.024873 | 0.044093 | 0.072357 | 0.012436 |
| **Ju-Fl** | 0.008479 | 0.00961 | 0.014132 | - | 0.004522 | 0.006218 | 0.002261 |
| **Fr** | 0.015828 | 0.027699 | 0.005653 | 0.002826 | - | 0.009045 | 0.019785 |
| **Fr-Fl** | 0.00961 | 0.023177 | 0.050876 | 0.003957 | 0.022046 | - | 0.007914 |
| **Fl** | 0.006218 | 0.002826 | 0.002261 | 0.000565 | 0.018089 | 0.005088 | - |
|  |  |  |  |  |  |  |  |
| **2011-2015** | **Ju** | **Ju-Fr** | **Ju-Fr-Fl** | **Ju-Fl** | **Fr** | **Fr-Fl** | **Fl** |
| **Ju** | - | 0.051913 | 0.059199 | 0.03643 | 0.015483 | 0.007286 | 0.010018 |
| **Ju-Fr** | 0.123862 | - | 0.060109 | 0.023679 | 0.014572 | 0.003643 | 0.003643 |
| **Ju-Fr-Fl** | 0.074681 | 0.040984 | - | 0.107468 | 0.010929 | 0.020036 | 0.010929 |
| **Ju-Fl** | 0.053734 | 0.01275 | 0.065574 | - | 0.000911 | 0.003643 | 0.003643 |
| **Fr** | 0.033698 | 0.02459 | 0.018215 | 0.002732 | - | 0.010929 | 0.010929 |
| **Fr-Fl** | 0.007286 | 0.009107 | 0.027322 | 0.007286 | 0.004554 | - | 0.003643 |
| **Fl** | 0.010018 | 0.001821 | 0.002732 | 0.005464 | 0.002732 | 0.001821 | - |

**References**

Madsen, J., Cracknell, G. & Fox (Eds.), A. D. (1999) *Goose populations of the western Palearctic. A review of status and distribution,* Wetlands International, Wageningen, The Netherlands, National Environmental research institute, Rönde, Denmark, Wetlands International Publ. No. 48.
